# COVID-19 pandemic: study on simple, easy, and practical relaxation techniques while wearing medical protective equipment

**DOI:** 10.1017/S0033291720003220

**Published:** 2020-08-24

**Authors:** Huiqin Zhang, Aimin Li, Boheng Zhu, Yanyan Niu, Zheng Ruan, Lihong Liu, Xiaoling Gao, Kun Wang, Lu Yin, Mao Peng, Qing Xue, Haixia Leng, Baoquan Min, Qing Tian, Chunxue Wang, Yuan Yang, Zhou Zhu, Tianmei Si, Wei Li, Fangfang Shangguan, Xia Hong, Hong Chang, Haiqing Song, Dongning Li, Longbin Jia, Huiqing Dong, Yuping Wang, Fiammetta Cosci, Hongxing Wang

**Affiliations:** 1 Department of Ultrasound, Beijing Tiantan Hospital, Capital Medical University, Beijing, China; 2 Department of Respiratory and Critical Care Medicine, The First Hospital of Shanxi Medical University, Taiyuan, China; 3 Department of Psychology, University of Bologna, Bologna, Italy; 4 Department of Orthopedics, Jincheng People's Hospital, Shanxi Medical University, Jincheng, China; 5 Division of Neuropsychiatry and Psychosomatics, Department of Neurology, Xuanwu Hospital, Capital Medical University, Beijing, China; 6 Department of Neurology, Ningcheng Center Hospital, Ningcheng, Inner Mongolia, China; 7 Department of Respiratory and Critical Care Medicine, The Second Hospital of Shanxi Medical University, Taiyuan, China; 8 Department of Neurology, Beijing Puren Hospital, Beijing, China; 9 Medical Research & Biometrics Center, Fuwai Hospital, National Center for Cardiovascular Diseases, Peking Union Medical College & Chinese Academy of Medical Sciences, Beijing, China; 10 Beijing Institute of Brain Disorders, Capital Medical University, Beijing, China; 11 Department of Neuropsychiatry and Behavioral Neurology and Clinical Psychology Center, Beijing Tiantan Hospital, Capital Medical University, Beijing, China; 12 Department of Psychiatry, Tongji Hospital, Tongji Medical College, Huazhong University of Science and Technology, Wuhan, China; 13 Peking University Sixth Hospital, Institute of Mental Health, National Clinical Research Center for Mental Health Disorders & Key Laboratory of Mental Health, Ministry of Health, Peking University, Beijing, China; 14 Department of Neurology, The Third People's Hospital of Chengdu, Chengdu, China; 15 School of Psychology, Capital Normal University, Beijing, China; 16 Department of Neurology, Xiyuan Hospital, China Academy of Chinese Medical Science, Beijing, China; 17 Department of Neurology, Jincheng People's Hospital, Shanxi Medical University, Jincheng, China; 18 Department of Health Sciences, University of Florence, Florence, Italy; 19 Beijing Psychosomatic Disease Consultation Center, Xuanwu Hospital, Capital Medical University, Beijing, China

**Keywords:** Coronavirus, COVID-19 pandemic, Health worker, Medical protective equipment, Relaxation

## Abstract

**Background:**

No studies have reported on how to relieve distress or relax in medical health workers while wearing medical protective equipment in coronavirus disease 2019 (COVID-19) pandemic. The study aimed to establish which relaxation technique, among six, is the most feasible in first-line medical health workers wearing medical protective equipment.

**Methods:**

This was a two-step study collecting data with online surveys. Step 1: 15 first-line medical health workers were trained to use six different relaxation techniques and reported the two most feasible techniques while wearing medical protective equipment. Step 2: the most two feasible relaxation techniques revealed by step 1 were quantitatively tested in a sample of 65 medical health workers in terms of efficacy, no space limitation, no time limitation, no body position requirement, no environment limitation to be done, easiness to learn, simplicity, convenience, practicality, and acceptance.

**Results:**

Kegel exercise and autogenic relaxation were the most feasible techniques according to step 1. In step 2, Kegel exercise outperformed autogenic relaxation on all the 10 dimensions among the 65 participants while wearing medical protective equipment (efficacy: 24 *v*. 15, no space limitation: 30 *v*. 4, no time limitation: 31 *v*. 4, no body position requirement: 26 *v*. 4, no environment limitation: 30 *v*. 11, easiness to learn: 28 *v*. 5, simplicity: 29 *v*. 7, convenience: 29 *v*. 4, practicality: 30 *v*. 14, acceptance: 32 *v*. 6).

**Conclusion:**

Kegel exercise seems a promising self-relaxation technique for first-line medical health workers while wearing medical protective equipment among COVID-19 pandemic.

## Introduction

The coronavirus disease 2019 (COVID-19) pandemic is ongoing globally (World Health Organization, 2020). Medical health workers, including medical doctors and nurses, are directly in contact with and treat COVID-19 patients (Zhang, Wang, et al., 2020). They are at a high chance of being infected (Chou et al., 2020; Givi et al., 2020), exposed to long and protracting work time (Zhang, Wang, et al., 2020), and have high levels of distress (Zhang, Wang, et al., 2020). Thus, they are at risk of burnout (Aronsson et al., 2017; Zhang, Wang, et al., 2020). Identifying a technique that might help them in managing such distress might be helpful to favor their working activity and maintain their mind−body balance. Relaxation techniques, among the others, seem promising (Pospos et al., 2018). Simple, easy, and practical methods are preferable (Zhang, Wang, et al., 2020), since medical health workers work wearing medical protective equipment which limits their movement, environmental management, space, and body position.

Relaxation is an individual feeling of calm and ease without tension in body and mind, which occurs minimizing neurological arousal (i.e. a feeling of being physically relaxed) and empowering enthusiastic emotions (such as feeling mentally rested, quiet, cheerful, and restored) (Klainin-Yobas, Oo, Suzanne Yew, & Lau, 2015). Many techniques can produce relaxation. They include abdominal breathing (Chen, Huang, Chien, & Cheng, 2017), yoga (Pascoe, Thompson, & Ski, 2017), autogenic relaxation (Hashim & Hanafi Ahmad Yusof, 2011), progressive muscle relaxation (Hashim & Hanafi Ahmad Yusof, 2011; Ozgundondu & Gok Metin, 2019), Kegel exercise (Huang & Chang, 2020), physical exercise (Silva et al., 2019; Yang & Chen, 2018), listening to music (Diri, Çetinkaya, & Gül, 2019), tai chi (Wang et al., 2014), guided imagery (Flynn, Jones, & Ausderau, 2016), biofeedback (Aritzeta et al., 2017), cognitive behavior therapy (Buhrman et al., 2015), and mindfulness therapy (Spadaro & Hunker, 2016), etc. Relaxation not only mitigates negative emotions but also enhances physical and mental well-being and relieves the feeling of stress and muscular tension (Hashim & Hanafi Ahmad Yusof, 2011; Klainin-Yobas et al., 2015; MacSween, Lorrimer, van Schaik, Holmes, & van Wersch, 2018; Ozgundondu & Gok Metin, 2019).

As of today, many papers have focused on the emergence of psychological distress and/or mental health in medical health workers during the pandemic (Bao, Sun, Meng, Shi, & Lu, 2020; D'Agostino, Demartini, Cavallotti, & Gambini, 2020; Kang et al., 2020; Liu et al., 2020; Walton, Murray, & Christian, 2020; Zhang, Yang, et al., 2020), but no studies reported on how to relieve such distress via a simple, easy, and practical relaxation technique to be used while wearing medical protective equipment (which cannot be removed during work time). This was the aim of this study. Medical staff who supported hospitals in Wuhan among the pandemic was tested to reach the goal.

## Methods

### Design, participants, and procedure

**Fig. 1.**
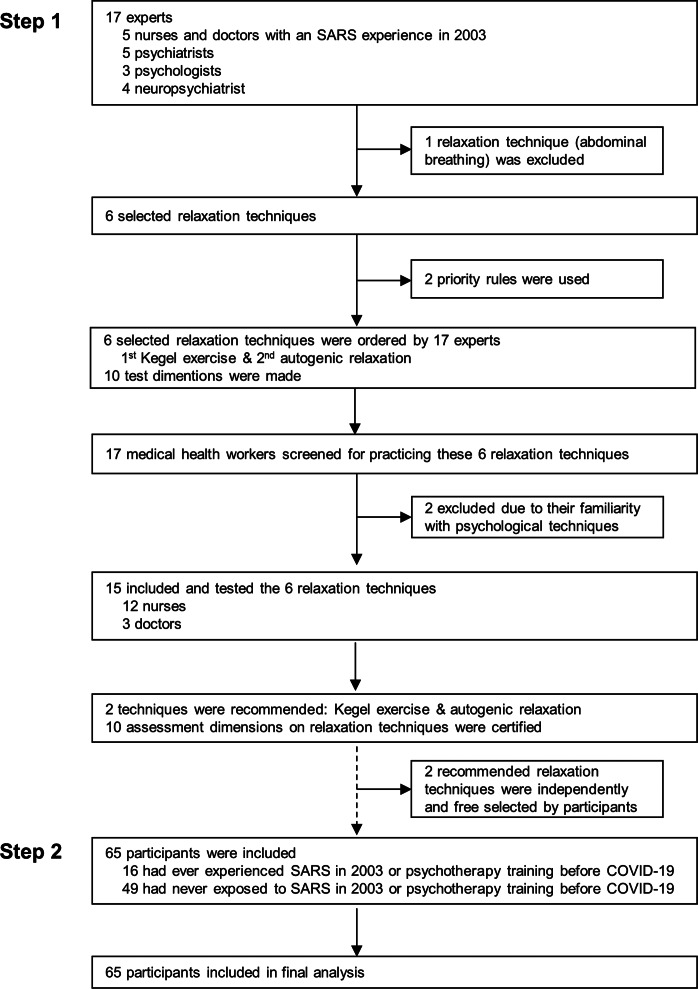
Study profile.

A two-step study was conducted (Fig. 1). All participants were 18−60 years and agreed with enrollment in the study. At step 1, a qualitative interview and a quantitative questionnaire survey on common possible relaxation techniques used by medical health workers were proposed to 17 experts (i.e. five nurses and doctors as first-line medical health workers who had severe acute respiratory syndrome − SARS experience in 2003, five psychiatrists, three psychologists, and four neuropsychiatrists who have expertise in relaxation techniques in their clinical settings). Six relaxation techniques were selected: autogenic relaxation (i.e. imagining previous peaceful places followed by developing an awareness of physical sensations) (Hashim & Hanafi Ahmad Yusof, 2011), Kegel exercise (i.e. pelvic-floor exercise which involves repeatedly contracting and relaxing the muscles that form part of the pelvic floor) (Huang & Chang, 2020), progressive muscle relaxation (i.e. tensing and relaxing the muscle groups, typically accompanied by deep breathing) (Hashim & Hanafi Ahmad Yusof, 2011; Ozgundondu & Gok Metin, 2019), guided imagery (i.e. controlling breathing and visualizing a soothing image) (Flynn et al., 2016), listening to music (Diri et al., 2019), and aerobic exercises (i.e. repetitive movement, such as walking, running, dancing, etc.) (Yang & Chen, 2018). Because COVID-19 is a disease dominantly transmitted through the respiratory system (Zhang, Li, Zhang, Wang, & Molina, 2020), abdominal breathing was not listed as one of the recommended techniques by our experts. Then, 15 medical health workers who had never experienced psychotherapy training and supporting Wuhan of Hubei Province from Inner Mongolia, Beijing, and Shanxi were recruited from January 21 to 25, 2020, and trained to use these six relaxation techniques. Thereafter, participants were qualitatively interviewed via telephone interviews, and data were quantitatively collected via a survey created *ad hoc* assessing several dimensions of relaxation technique to identify the most feasible one. As a result, autogenic relaxation and Kegel exercise were recommended by the 15 first-line medical health workers for their working hours.

At step 2, 65 participants (39 of Shanxi, 10 in Beijing, 17 of Inner Mongolia), who were at work from April 10 to May 10, 2020, volunteered to practice autogenic relaxation and Kegel exercise. The instructions for these relaxation techniques were published online at the official WeChat public account of Department of Neurology of Xuanwu Hospital, Capital Medical University, and thus made available for all first-line medical staff. In addition, the online link was also directly sent via WeChat to the 65 volunteers to ensure that they received it and read this material before arriving at Hubei.

Two days after having received the material and being asked to practice autogenic relaxation and/or Kegel exercise for the following 70 days at work, the 65 participants were invited to fill in the online survey created *ad hoc* evaluating the proposed dimensions of relaxation technique in step 1, aiming to identify the most feasible one. Data were collected anonymously via the Wenjuanxing platform (https://www.wjx.cn/m/73948651.aspx).

### Instruments

The online survey was used at step 1 and at step 2 allowed to collect sex, age, marital status, education, psychotherapy before the pandemic COVID-19, participation in the campaign against SARS in 2003. In addition, 10 dimensions, including efficacy, no space, no time, no body position, no environment limitation; easiness to learn, simplicity, convenience, practicality, and acceptance were assessed referring to autogenic relaxation or Kegel exercise. Each dimension was rated on an analogue scale ranging from 0 (not at all) to 10 (maximum). Each subject could use both or one single relaxation technique, according to his/her preference, and was asked to rate each technique that they had used.

In addition, in order to verify which relaxation method was best suited for medical staffs while wearing medical protective equipment or in leisure time in Wuhan of China, the following questions were formulated: ‘which relaxation technique did you prefer while wearing medical protective equipment?’ and ‘which relaxation technique did you prefer during your leisure time among these six relaxation techniques?’.

### Statistical analysis

Frequencies were calculated for each dimension for each relaxation technique. Median, interquartile range (IQR), mean, and standard deviation (s.d.) were used to further describe each dimension. The percentage of the preferred relaxation method during leisure time was also determined.

The previous experience on SARS or psychotherapy training before COVID-19 pandemic may affect participants’ choices on the two recommended relaxation techniques, so Chi-square tests were used to compare those who were previously exposed to SARS or psychotherapy and those who had never experienced SARS or psychotherapy concerning the 10 dimensions assessed for autogenic relaxation and Kegel exercise.

## Results

### Results of step 1

Based on the qualitative interviews, the 17 experts agreed that ‘easily learning by their reading directions on relaxation techniques’ was of the most priority. Based on this, six relaxation techniques were identified: Kegel exercise (17/17), autogenic relaxation (17/17), progressive muscle relaxation (14/17), guided imagery (11/17), aerobic exercise (3/17), listening to music (1/17). Secondly, all experts agreed that ‘no need to pour more energy and attention’ was another priority. Based on this, Kegel exercise (17/17), autogenic relaxation (16/17), progressive muscle relaxation (11/15), guided imagery (10/17), listening to music (2/17), aerobic exercise (1/17), were selected.

The six relaxation techniques were tested among 15 of 17 participants while supporting medical services and wearing medical protective equipment; the two subjects who had experienced psychotherapy were excluded, due to their familiarity with specific psychological techniques. According to the choice of the 15 subjects, Kegel exercise (15/15), autogenic relaxation (15/15), guided imagery (10/15), progressive muscle relaxation (8/15), aerobic exercise (3/15), listening to music (0/15) were selected. In working condition, aerobic exercise was not preferred as 15 participants worried that aerobic exercise might have a risk of breaking medical protective equipment; likewise, listening to music needed specific tools and was not preferable. However, they thought that these two relaxation methods might be good in their leisure time. All recruited participants considered that Kegel exercise and autogenic relaxation were feasible relaxation techniques while wearing medical protective equipment, and 10 dimensions of the assessment of the relaxation techniques were proposed (Fig. 1). The steps on Kegel exercises applied in our study were: 1. To find your pelvic muscles. Assume you are trying to prevent traveling gas. If you've recognized the correct muscles, you'll notice the tightening more in the back of the pelvic region than the front. 2. To flex pelvic floor muscles for 3 to 5 s. 3. To relax pelvic floor muscles for 3 to 5 s. 4. To replicate the contraction−relaxation pattern 8–10 times. After you have grasped the steps, you can practice freely anytime and anywhere without the restriction of position for experiencing self-relaxation. However, it should be noted that, do not habituate Kegel exercises to begin and discontinue your urine flow; do not contract the muscles of your abdomen, thighs, or buttocks; and keep freely breathing among the contraction−relaxation exercises.

### Results of step 2

Data from 65 subjects (41 female, 24 male) with a mean age of 38.0 ± 4.1 years (minimum: 32; maximum: 55 years) were collected. They confirmed that they freely and independently tried to select and apply these relaxation techniques to relax when they were not in the handling of the patient's affairs in their medical protective equipment, and admitted that these relaxation techniques to some extent benefited their reducing stress. All participants anonymously completed the online survey. Of them, six were single, 59 married; 19 were doctors and 46 nurses; all had more than 14-year educational level; three had SARS experience in 2003, and 12 were on psychotherapy before the pandemic. Their working experience was of 15.7 ± 3.9 years (from 7 to 30 years).

**Fig. 2.**
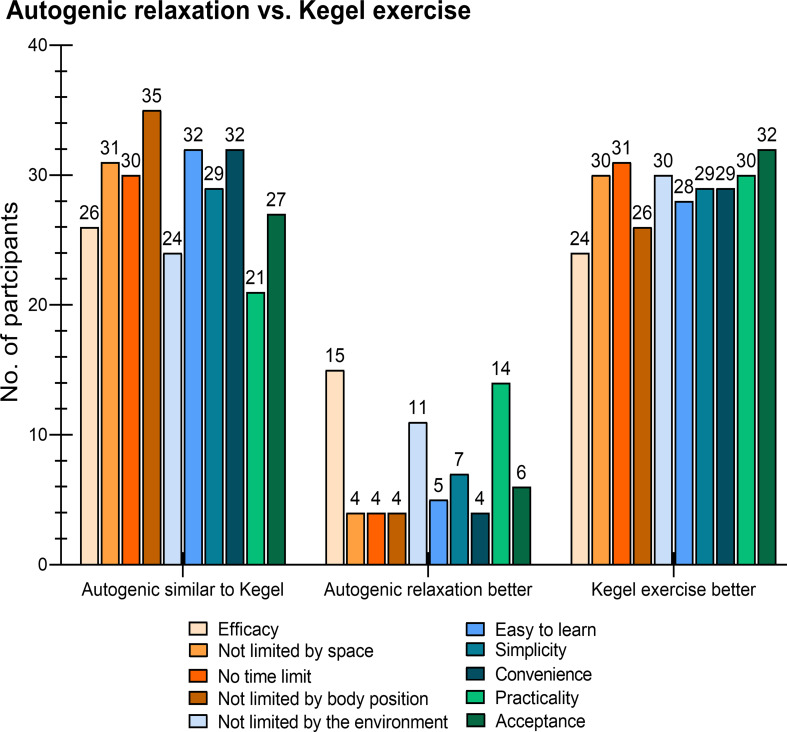
Frequencies for each dimension of autogenic relaxation and Kegel exercise in 65 participants.

**Fig. 3.**
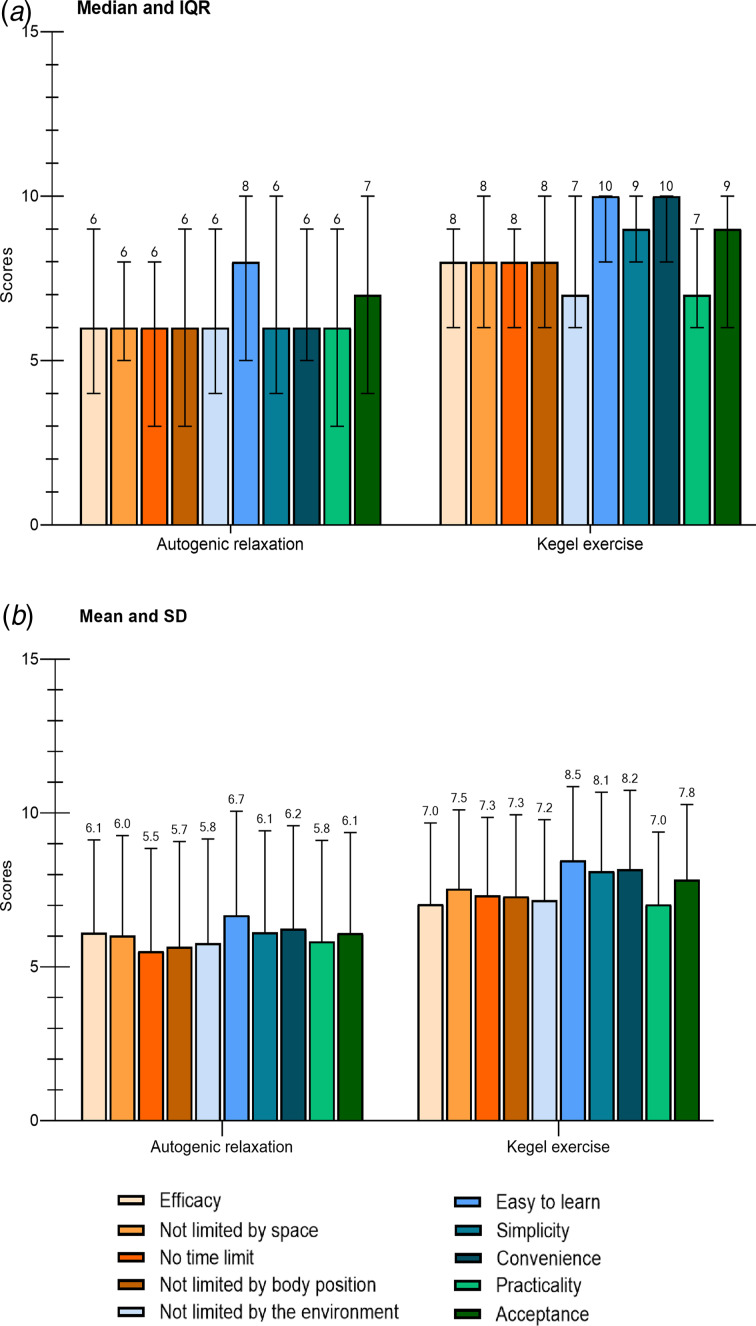
Scores on 10 dimensions of (a) autogenic relaxation and (b) Kegel exercise in 65 participants. IQR = interquartile range; s.d. = standard deviation.

During working time and while wearing medical protective equipment, more participants considered Kegel exercise better than autogenic relaxation on the 10 dimensions (efficacy: 24 *v*. 15, no space limitation: 30 *v*. 4, no time limitation: 31 *v*. 4, no body position requirement: 26 *v*. 4, no environment limitation to be done: 30 *v*. 11, easiness to learn: 28 *v*. 5, simplicity: 29 *v*. 7, convenience: 29 *v*. 4, practicality: 30 *v*. 14, acceptance: 32 *v*. 6, Fig. 2), and the scores on the 10 dimensions of Kegel exercise was higher than that of autogenic relaxation (Fig. 3*a* and 3*b*). On the contrary, during leisure time, the preferred relaxation technique was autogenic relaxation (46.2%), followed by listening to music (35.2%), and physical exercise (16.9%).

We further analyzed differences in 10 dimensions of the two selected relaxation techniques between those who had never experienced SARS or psychotherapy before COVID-19 (group 1, *n* = 49) and those who had exposed to SARS or psychotherapy (group 2, *n* = 16). Both groups considered that Kegel exercise and autogenic relaxation were not statistically different on all tested dimensions (Table 1).

**Table 1.** Differences on 10 dimensions of autogenic relaxation and Kegel exercise between participants who had never experienced SARS or psychotherapy training before COVID-19 (group 1) and those who had ever exposed to SARS or psychotherapy (group 2)<TE: Please take care to set shading in the table.>

	Autogenic similar to Kegel		Autogenic relaxation better		Kegel exercise better		*p* value
	Group 1 (*n* = 49)	group 2 (*n* = 16)	Group 1 (*n* = 49)	Group 2 (*n* = 16)	Group 1 (*n* = 49)	Group 2 (*n* = 16)	
Effectiveness	20 (41%)	6 (38%)	9 (18%)	6 (38%)	20 (41%)	4 (25%)	0.62
Not limited by space	23 (47%)	8 (50%)	4 (8%)	0	22 (45%)	8 (50%)	0.94
No time limit	19 (39%)	11 (69%)	4 (8%)	0	26 (53%)	5 (31%)	0.07
Not limited by body position	24 (49%)	11 (69%)	4 (8%)	0	21 (43%)	5 (31%)	0.26
Not limited by the environment	16 (33%)	8 (50%)	11 (22%)	0	22 (45%)	8 (50%)	0.10
Easy to learn	21 (43%)	11 (69%)	5 (10%)	0	23 (47%)	5 (31%)	0.14
Simplicity	21 (43%)	8 (50%)	5 (10%)	2 (13%)	23 (47%)	6 (38%)	0.55
Convenience	21 (43%)	11 (69%)	4 (8%)	0	24 (49%)	5 (31%)	0.12
Practicality	16 (33%)	5 (31%)	8 (16%)	6 (38%)	25 (51%)	5 (31%)	0.47
Acceptance	19 (39%)	8 (50%)	3 (6%)	3 (19%)	27 (55%)	5 (31%)	0.20

COVID-19, coronavirus disease 2019; SARS, severe acute respiratory syndrome.

## Discussion

At present, there is a lack of solid guidelines for favoring relaxation in medical health workers while wearing medical protective equipment, although this might be a helpful strategy to reduce distress and burnout risk in medical health staff.

The present study showed that Kegel exercise was an effective, simple, practical method of self-relaxation by medical health workers when they are at work and wear medical protective equipment. The main reasons of such a preference were: (1) more participants thought Kegel exercise was better than autogenic relaxation from 10 assessment dimensions; (2) scores on 10 dimensions on Kegel exercise were higher than those on autogenic relaxation; (3) Kegel exercise is not limited by body position, including sitting, standing, or walking. (4) Kegel exercise is a traditional method of strengthening the body, involving traditional Chinese medicine (Tsai et al., 1995). On the contrary, during leisure time, autogenic relaxation was firstly preferred. Additionally, listening to music and physical exercise were self-relaxation techniques followed by autogenic relaxation used by medical staff during leisure time. Our results indicate that autogenic relaxation, listening to music, and physical exercise do have a role in reducing stress and protect brain and body proposed by the previously reported studies on other populations (Cervellin & Lippi, 2011; Klainin-Yobas et al., 2015; Nabi et al., 2013; Pospos et al., 2018; Seo et al., 2018; Silva et al., 2019; Yang & Chen, 2018). To be noted, participants of the study often stayed in hotel rooms in Hubei during their leisure time due to COVID-19's airborne transmission via aerosol and droplets (Izzetti, Nisi, Gabriele, & Graziani, 2020; Zhang, Li, et al., 2020). This might have influenced their preferences. Furthermore, a broader approach such as sleep hygiene, social support, and facilitation from employer or organizer might be needed to reduce their stress when medical health workers were in their leisure time (Theorell, 2020). Moreover, Ost's applied relaxation, although not our relaxation methods in the study, might be a strategy to rapidly relax in an increasingly stressful situation (Hayes-Skelton, Roemer, Orsillo, & Borkovec, 2013; Ost, 1987; 1988).

Until now, few previous studies have reported how medical staff relieves their distress at work and during leisure times (Aronsson et al., 2017; Pospos et al., 2018). Work environment can induce medical staff burnout symptoms (Aronsson et al., 2017), and seven web-based instruments and mobile utilizations were devised to lessen burnout, negative emotion, and even suicide among healthcare students and professionals (Pospos et al., 2018). However, no studies on relaxation techniques suitable for medical staff while wearing medical protective equipment are available.

This is the first study to explore the possible simple self-relaxation method when people were in a strictly limited working environment. Our study sample of step 2 was strictly limited to medical health workers who volunteered to support medical service in Wuhan during the epidemic. Therefore, they were a typical representative group in a special environment, such as being a contracted condition. The present study has, however, limitations. First, a cross-sectional design was used, a pre- and -post design with specific questionnaires on the assessment of anxiety and tension, which might provide additional and stronger information. Second, except for the recruited 65 participants, it is unknown how many subjects who visited or accepted these recommended relaxation techniques and the online survey on their evaluations about these techniques. Third, the dimensions were assessed via a self-rated analogue scale, a clinician-rating method might be helpful to collect data more objectively.

## Conclusions and implications

Kegel exercise was preferred for first-line medical health workers at working, while autogenic relaxation was preferred during leisure time among the pandemic. There is apparently room for proposing simple, easy, and practical self-relaxation techniques to first-line medical health workers while they must wear medical protective equipment in order to reduce their level of stress.

## References

[ref2] Aritzeta, A., Soroa, G., Balluerka, N., Muela, A., Gorostiaga, A., & Aliri, J. (2017). Reducing anxiety and improving academic performance through a biofeedback relaxation training program. Applied Psychophysiology and Biofeedback, 42(3), 193–202. doi:10.1007/s10484-017-9367-z. 28623467

[ref3] Aronsson, G., Theorell, T., Grape, T., Hammarstrom, A., Hogstedt, C., Marteinsdottir, I., … Hall, C. (2017). A systematic review including meta-analysis of work environment and burnout symptoms. BMC Public Health, 17(1), 264. doi:10.1186/s12889-017-4153-7. 28302088 PMC5356239

[ref4] Bao, Y., Sun, Y., Meng, S., Shi, J., & Lu, L. (2020). 2019-nCoV epidemic: Address mental health care to empower society. The Lancet, 395(10224), e37–e38. doi:10.1016/s0140-6736(20)30309-3. PMC7133594 32043982

[ref5] Buhrman, M., Syk, M., Burvall, O., Hartig, T., Gordh, T., & Andersson, G. (2015). Individualized guided internet-delivered cognitive-behavior therapy for chronic pain patients with comorbid depression and anxiety: A randomized controlled trial. The Clinical Journal of Pain, 31(6), 504–516. doi:10.1097/ajp.0000000000000176. 25380222

[ref6] Cervellin, G., & Lippi, G. (2011). From music-beat to heart-beat: A journey in the complex interactions between music, brain and heart. European Journal of Internal Medicine, 22(4), 371–374. doi:10.1016/j.ejim.2011.02.019. 21767754

[ref7] Chen, Y. F., Huang, X. Y., Chien, C. H., & Cheng, J. F. (2017). The effectiveness of diaphragmatic breathing relaxation training for reducing anxiety. Perspectives in Psychiatric Care, 53(4), 329–336. doi:10.1111/ppc.12184. 27553981

[ref8] Chou, R., Dana, T., Buckley, D. I., Selph, S., Fu, R., & Totten, A. M. (2020). Epidemiology of and risk factors for coronavirus infection in health care workers: a living rapid review. Annals of Internal Medicine, 173(2), 120–136. doi:10.7326/m20-1632. 32369541 PMC7240841

[ref9] D'Agostino, A., Demartini, B., Cavallotti, S., & Gambini, O. (2020). Mental health services in Italy during the COVID-19 outbreak. The Lancet Psychiatry, 7(5), 385–387. doi:10.1016/s2215-0366(20)30133-4. 32353266 PMC7185925

[ref10] Diri, M. A., Çetinkaya, F., & Gül, M. (2019). The effects of listening to music on anxiety, pain, and satisfaction during urodynamic study: A randomized controlled trial. Urologia Internationalis, 103(4), 444–449. doi:10.1159/000502298. 31408870

[ref11] Flynn, T. A., Jones, B. A., & Ausderau, K. K. (2016). Guided imagery and stress in pregnant adolescents. American Journal of Occupational Therapy, 70(5), 7005220020p1–7005220020p7. doi:10.5014/ajot.2016.019315. 27548866

[ref12] Givi, B., Schiff, B. A., Chinn, S. B., Clayburgh, D., Iyer, N. G., Jalisi, S., … Davies, L. (2020). Safety recommendations for evaluation and surgery of the head and neck during the COVID-19 pandemic [published online ahead of print, 2020 Mar 31]. JAMA Otolaryngology-- Head & Neck Surgery. doi:10.1001/jamaoto.2020.0780. 32232423

[ref13] Hashim, H. A., & Hanafi Ahmad Yusof, H. (2011). The effects of progressive muscle relaxation and autogenic relaxation on young soccer players' mood states. Asian Journal of Sports Medicine, 2(2), 99–105. doi:10.5812/asjsm.34786. 22375225 PMC3289204

[ref14] Hayes-Skelton, S. A., Roemer, L., Orsillo, S. M., & Borkovec, T. D. (2013). A contemporary view of applied relaxation for generalized anxiety disorder. Cognitive Behavioural Therapy, 42(4), 292–302. doi:10.1080/16506073.2013.777106. PMC3797858 23731329

[ref15] Huang, Y. C., & Chang, K. V. (2020). Kegel Exercises. [Updated 2020 May 29]. Available at https://www.ncbi.nlm.nih.gov/books/NBK555898/.

[ref16] Izzetti, R., Nisi, M., Gabriele, M., & Graziani, F. (2020). COVID-19 Transmission in dental practice: Brief review of preventive measures in Italy. Journal of Dental Research. 99(9), 1030–1038. doi:10.1177/0022034520920580. 32302257

[ref17] Kang, L., Li, Y., Hu, S., Chen, M., Yang, C., Yang, B. X., … Liu, Z. (2020). The mental health of medical workers in Wuhan, China dealing with the 2019 novel coronavirus. The Lancet Psychiatry, 7(3), e14. doi:10.1016/s2215-0366(20)30047-x. 32035030 PMC7129673

[ref18] Klainin-Yobas, P., Oo, W. N., Suzanne Yew, P. Y., & Lau, Y. (2015). Effects of relaxation interventions on depression and anxiety among older adults: A systematic review. Aging & Mental Health, 19(12), 1043–1055. doi:10.1080/13607863.2014.997191. 25574576

[ref19] Liu, S., Yang, L., Zhang, C., Xiang, Y. T., Liu, Z., Hu, S., & Zhang, B. (2020). Online mental health services in China during the COVID-19 outbreak. The Lancet Psychiatry, 7(4), e17–e18. doi:10.1016/S2215-0366(20)30077-8. 32085841 PMC7129099

[ref20] MacSween, A., Lorrimer, S., van Schaik, P., Holmes, M., & van Wersch, A. (2018). A randomised crossover trial comparing Thai and Swedish massage for fatigue and depleted energy. Journal of Bodywork and Movement Therapies, 22(3), 817–828. doi:10.1016/j.jbmt.2017.09.014. 30100318

[ref21] Nabi, H., Kivimäki, M., Batty, G. D., Shipley, M. J., Britton, A., Brunner, E. J., … Singh-Manoux, A. (2013). Increased risk of coronary heart disease among individuals reporting adverse impact of stress on their health: The Whitehall II prospective cohort study. European Heart Journal, 34(34), 2697–2705. doi:10.1093/eurheartj/eht216. 23804585 PMC3766148

[ref22] Ost, L. G. (1987). Applied relaxation: Description of a coping technique and review of controlled studies. Behaviour Research and Therapy, 25(5), 397–409. doi:10.1016/0005-7967(87)90017-9. 3318800

[ref23] Ost, L. G. (1988). Applied relaxation vs progressive relaxation in the treatment of panic disorder. Behaviour Research and Therapy, 26(1), 13–22. doi:10.1016/0005-7967(88)90029-0. 3277613

[ref24] Ozgundondu, B., & Gok Metin, Z. (2019). Effects of progressive muscle relaxation combined with music on stress, fatigue, and coping styles among intensive care nurses. Intensive and Critical Care Nursing, 54, 54–63. doi:10.1016/j.iccn.2019.07.007. 31371164

[ref25] Pascoe, M. C., Thompson, D. R., & Ski, C. F. (2017). Yoga, mindfulness-based stress reduction and stress-related physiological measures: A meta-analysis. Psychoneuroendocrinology, 86, 152–168. doi:10.1016/j.psyneuen.2017.08.008. 28963884

[ref26] Pospos, S., Young, I. T., Downs, N., Iglewicz, A., Depp, C., Chen, J. Y., … Zisook, S. (2018). Web-based tools and mobile applications to mitigate burnout, depression, and suicidality among healthcare students and professionals: A systematic review. Academic Psychiatry, 42(1), 109–120. doi:10.1007/s40596-017-0868-0. 29256033 PMC5796838

[ref27] Seo, E., Hong, E., Choi, J., Kim, Y., Brandt, C., & Im, S. (2018). Effectiveness of autogenic training on headache: A systematic review. Complementary Therapies in Medicine, 39, 62–67. doi:10.1016/j.ctim.2018.05.005. 30012394

[ref28] Silva, L. A. D., Tortelli, L., Motta, J., Menguer, L., Mariano, S., Tasca, G., … Silveira, P. C. L. (2019). Effects of aquatic exercise on mental health, functional autonomy and oxidative stress in depressed elderly individuals: A randomized clinical trial. Clinics *(*Sao Paulo), 74, e322. doi:10.6061/clinics/2019/e322. 31271585 PMC6585867

[ref29] Spadaro, K. C., & Hunker, D. F. (2016). Exploring the effects of an online asynchronous mindfulness meditation intervention with nursing students on stress, mood, and cognition: A descriptive study. Nurse Education Today, 39, 163–169. doi:10.1016/j.nedt.2016.02.006. 27006051

[ref30] Theorell, T. (2020). COVID-19 and working conditions in health care. Psychotherapy and Psychosomatics, 89(4), 193–194. doi:10.1159/000507765. 32299083 PMC7206352

[ref31] Tsai, T. J., Lai, J. S., Lee, S. H., Chen, Y. M., Lan, C., Yang, B. J., & Chiang, H. S. (1995). Breathing-coordinated exercise improves the quality of life in hemodialysis patients. Journal of the American Society of Nephrology, 6(5), 1392–1400. 8589314 10.1681/ASN.V651392

[ref31a] Walton, M., Murray, E., & Christian, M. D. (2020). Mental health care for medical staff and affiliated healthcare workers during the COVID-19 pandemic. Eur Heart J Acute Cardiovasc Care, 9(3), 241–247. doi:10.1177/2048872620922795. 32342698 PMC7189614

[ref32] Wang, F., Lee, E. K., Wu, T., Benson, H., Fricchione, G., Wang, W., & Yeung, A. S. (2014). The effects of tai chi on depression, anxiety, and psychological well-being: A systematic review and meta-analysis. International Journal of Behavioral Medicine, 21(4), 605–617. doi:10.1007/s12529-013-9351-9. 24078491

[ref33] World Health Organization. (2020). Coronavirus disease (COVID-19): Situation Report-145. Available at https://www.who.int/docs/default-source/coronaviruse/situation-reports/20200613-covid-19-sitrep-145.pdf?sfvrsn=bb7c1dc9_2.

[ref34] Yang, C. L., & Chen, C. H. (2018). Effectiveness of aerobic gymnastic exercise on stress, fatigue, and sleep quality during postpartum: A pilot randomized controlled trial. International Journal of Nursing Studies, 77, 1–7. doi:10.1016/j.ijnurstu.2017.09.009. 28950158

[ref35] Zhang, R., Li, Y., Zhang, A. L., Wang, Y., & Molina, M. J. (2020). Identifying airborne transmission as the dominant route for the spread of COVID-19. Proceedings of the National Academy of Sciences of the United States of America, 117(26), 14857–14863. doi: 10.1073/pnas.2009637117. 32527856 PMC7334447

[ref36] Zhang, W. R., Wang, K., Yin, L., Zhao, W. F., Xue, Q., Peng, M., … Wang, H. X. (2020). Mental health and psychosocial problems of medical health workers during the COVID-19 epidemic in China. Psychotherapy and Psychosomatics, 89(4), 242–250. doi:10.1159/000507639. 32272480 PMC7206349

[ref37] Zhang, C., Yang, L., Liu, S., Ma, S., Wang, Y., Cai, Z., … Zhang, B. (2020). Survey of insomnia and related social psychological factors among medical staff involved in the 2019 novel coronavirus disease outbreak. Frontiers in Psychiatry, 11, 306. doi:10.3389/fpsyt.2020.00306. 32346373 PMC7171048

